# Continuous Monitoring of Vital Signs Using Wearable Devices on the General Ward: Pilot Study

**DOI:** 10.2196/mhealth.7208

**Published:** 2017-07-05

**Authors:** Mariska Weenk, Harry van Goor, Bas Frietman, Lucien JLPG Engelen, Cornelis JHM van Laarhoven, Jan Smit, Sebastian JH Bredie, Tom H van de Belt

**Affiliations:** 1 Radboud University Medical Center Department of Surgery Nijmegen Netherlands; 2 Radboud University Medical Center Radboud REshape Innovation Center Nijmegen Netherlands; 3 Radboud University Medical Center Department of Internal Medicine Nijmegen Netherlands

**Keywords:** remote sensing technology, vital signs, wireless technology, continuous monitoring

## Abstract

**Background:**

Measurement of vital signs in hospitalized patients is necessary to assess the clinical situation of the patient. Early warning scores (EWS), such as the modified early warning score (MEWS), are generally calculated 3 times a day, but these may not capture early deterioration. A delay in diagnosing deterioration is associated with increased mortality. Continuous monitoring with wearable devices might detect clinical deterioration at an earlier stage, which allows clinicians to take corrective actions.

**Objective:**

In this pilot study, the feasibility of continuous monitoring using the ViSi Mobile (VM; Sotera Wireless) and HealthPatch (HP; Vital Connect) was tested, and the experiences of patients and nurses were collected.

**Methods:**

In this feasibility study, 20 patients at the internal medicine and surgical ward were monitored with VM and HP simultaneously for 2 to 3 days. Technical problems were analyzed. Vital sign measurements by nurses were taken as reference and compared with vital signs measured by both devices. Patient and nurse experiences were obtained by semistructured interviews.

**Results:**

In total, 86 out of 120 MEWS measurements were used for the analysis. Vital sign measurements by VM and HP were generally consistent with nurse measurements. In 15% (N=13) and 27% (N=23) of the VM and HP cases respectively, clinically relevant differences in MEWS were found based on inconsistent respiratory rate registrations. Connection failure was recognized as a predominant VM artifact (70%). Over 50% of all HP artifacts had an unknown cause, were self-limiting, and never took longer than 1 hour. The majority of patients, relatives, and nurses were positive about VM and HP.

**Conclusions:**

Both VM and HP are promising for continuously monitoring vital signs in hospitalized patients, if the frequency and duration of artifacts are reduced. The devices were well received and comfortable for most patients.

## Introduction

In hospitalized patients, vital signs are measured to assess the clinical situation of the patient and to identify clinical deterioration [1]. Monitoring of these vital signs is usually done by nurses, and includes blood pressure (BP), heart rate (HR), respiratory rate (RR), blood oxygen saturation, and core temperature. Early warning scores (EWS) are physiological track-and-trigger systems, which use a multiparameter or aggregate weighted scoring system that assists in detecting physiological changes and thereby identify patients at risk for further deterioration [2,3]. The modified early warning score (MEWS) is a commonly used and validated EWS system (see Multimedia Appendix 1) [4-6]. A higher MEWS is associated with admissions to the intensive care unit (ICU), cardiac arrest, and mortality [7-9]. Since the introduction of EWS, a trend was seen toward a decrease in unplanned admissions to the ICU and a decrease in hospital mortality [10-16]. Although the EWS provides relevant data on patients’ health status, the interval measurements may not capture early deterioration of vital signs [17], particularly during the night when clinical deterioration may remain undetected until the next day [18]. This could explain why the majority of the unplanned ICU admissions take place between 8 am and 4 pm [19]. Unplanned ICU admissions are associated with an increased mortality rate, a longer hospital stay [20-22], and a 60% increase in hospitalization costs [23]. Continuous monitoring of vital signs could be a useful tool to detect clinical deterioration in an earlier phase, which allows clinicians to take corrective interventions, particularly since subtle changes in vital signs often are present 8 to 24 hours before a life-threatening event such as ICU admission, cardiac arrest, and death [13,24-27]. Nowadays, wearable devices that facilitate remote continuous monitoring of vital signs exist [28]. These wireless devices could reduce patient discomfort due to fewer measurements by nurses [29-31], allow patient mobility [31], and might reduce workload for nurses [30]. Moreover, wearable devices are promising for safe patient transports between wards, the operating room, and the radiology department [32]. However, these devices are still underutilized in health care, even though they have been shown to be accurate [17,33], and may reduce costs [34]. Despite many potential advantages, wearable devices may have disadvantages regarding technical dysfunction and adverse psychological effects increasing anxiety of patients for disturbances of vital signs [33].

Recently, ViSi Mobile (VM; Sotera Wireless) and HealthPatch (HP; Vital Connect), two new devices approved by the US Food and Drug Administration (FDA) for wireless remote monitoring of vital signs, were introduced in health care. At present, little is known about the feasibility of continuous monitoring and experiences of patients and caregivers. The objective of this pilot study was to assess the technical feasibility of continuous monitoring with these new devices and to evaluate the experiences of patients and nurses with this method of monitoring on the general ward.

## Methods

### Setting and Recruitment

Patients hospitalized in the internal medicine and surgical ward of the Radboud University Medical Center were included between December 2014 and March 2015. All consecutively admitted patients were approached for participation if they were hospitalized for at least 48 hours, and MEWS measurements were ordered at least three times a day by their medical doctor. Patients had to be 18 years or older and able to speak, read, and understand the local language. At the internal medicine ward, both VM and HP were attached to the patient after signed informed consent was obtained. At the surgical ward, patients signed informed consent before an elective surgical procedure. Both devices were attached to the patients after surgery and arrival at the ward. Patients were excluded from further analyses if they unexpectedly participated for a duration shorter than 24 hours in the study. To determine the technical feasibility and practical usability, the two wearable devices were used to continuously measure vital signs in patients, which were compared with regular data collected in the same patients. Since a formal power calculation was not feasible due to the lack of preliminary data with these monitoring systems, a sample size of 20 was estimated to obtain sufficient data for analysis. After reviewing the study protocol, the institutional review board waived the need for formal review and approval (number 2014-1434).

### ViSi Mobile

**Figure 1:**
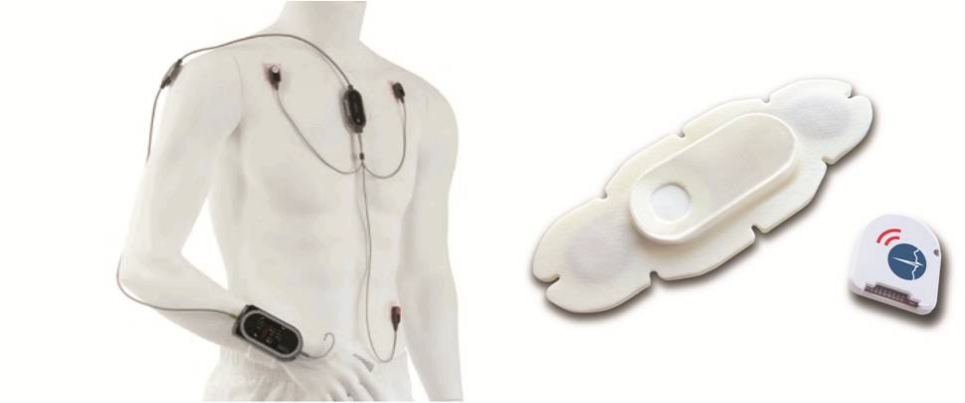
ViSi Mobile system (left) and HealthPatch (right).

The VM system has received Conformité Européenne (CE) mark and is FDA-cleared for continuously monitoring of 3- or 5-lead electrocardiogram (ECG), heart and pulse rate, blood oxygen saturation, RR, skin temperature, and BP (cuff-based and cuff-less on beat-to-beat basis; Figure 1). All vital signs are displayed on a patient-worn wrist device, which can be locked by an authentication code. This wrist device is connected to a thumb sensor, which measures blood oxygen saturation and BP. A chest sensor measures RR and skin temperature, and is connected with 3 or 5 ECG cables and sensors. In this pilot study, VM was wirelessly connected to a stand-alone Toughbook (Panasonic) pre-installed with VM software, from where the investigators received real-time insights on patients’ vital signs and where all the data were stored. This Toughbook also showed alarms as soon as vital signs dropped out of normal ranges. The VM wrist device was powered by rechargeable batteries, which needed to be replaced every 12 to 14 hours.

### HealthPatch

The HP consists of a reusable sensor and a disposable adhesive patch with 2 ECG electrodes at the bottom of the patch and a reusable sensor (see Figure 1). The HP has received CE mark and is FDA-cleared for continuous measurement of single-lead ECG, HR, heart rate variability (HRV), RR, skin temperature, body posture, fall detection, and activity. This small and lightweight patch can be attached to the patient’s chest, from where the data is transmitted to a mobile device (eg, mobile phone, via Bluetooth). Wi-Fi connection facilitates data transmission from the mobile device to a secured cloud server. The patch is powered by a coin-cell battery that lasts 3 to 4 days.

### Study Procedures

Patients gave verbal and written consent after being informed about the study protocol. Demographics including gender, age, reason for admission, and type of surgery were collected. At the surgical ward, VM and HP were attached to the patient after surgery and arrival at the ward. At the internal medicine ward, both devices were attached to the patient directly after signed informed consent was obtained. Vital signs were continuously measured during 2 or 3 days. This time frame was chosen to obtain enough vital sign data for analysis and to allow patients to get familiar with the devices. Nurses measured vital signs three times daily according to the protocol. Trained medical students additionally observed time-related vital signs monitored by VM at the Toughbook and HP on the cloud server. They marked the time points where vital signs were taken by the nurse and manually selected the results for vital signs measured by both devices at these time points for comparison. They also registered the cause and duration of technical problems and fixed them when necessary. In case of a VM alarm, the student warned the nurse. After 2 to 3 days, the enrolled patients and their relatives were interviewed about their experiences regarding continuous monitoring and both wearable devices. Nurses involved in the care of included patients were interviewed as well.

### Data Collection and Analysis

#### Technical Feasibility

All registered data from VM and HP were retrieved for analysis in the Statistical Package for the Social Sciences version 20.0 (SPSS, Inc). Data of both devices were compared with measurements by nurses at the same time points. For each variable, the accepted discrepancy between nurse measurements and both devices was determined, which are listed in Table 1. These thresholds were defined as the maximum possible discrepancy in vital signs between the nurse measurements and both devices that would not lead to a change in medical treatment. A difference in MEWS score of 1 point or more between the nurse measurements and both devices was defined as a clinically relevant difference. The MEWS scores were calculated using vital signs measured by the nurses, VM, and HP. As VM and HP did not measure all required vital signs to calculate the MEWS score, such as level of consciousness, these vital signs were taken from the electronic health records (EHR). Bland-Altman plots [35] were created to assess the agreement between MEWS measurements by nurses and corresponding values of VM and HP. All artifacts ≥1 minute were analyzed, since we reasoned that artifacts of less than one minute would not be clinically relevant for a patient’s situation. An artifact had occurred if no or an invalid value was recorded. Since trained medical students were not present all the time (primarily not during out-of-office hours), artifacts were divided into two groups, depending on the presence of a student.

**Table 1:** Accepted discrepancies between nurse measurements, ViSi Mobile, and HealthPatch.

Vital sign	Accepted discrepancy
Heart rate	5 beats/min
Respiratory rate	2 breaths/min
Oxygen saturation	2%
Temperature^a^	0.5˚C
Blood pressure	5 mm Hg
MEWS	1

^a^ViSi Mobile and HealthPatch measure skin temperature.

#### Practical Usability

User experiences were obtained by means of semistructured face-to-face interviews, after the patients had used the devices for 2 to 3 days. Patients’ relatives and nurses were also interviewed. Interviews lasted approximately 10 minutes and the following topics were discussed: feelings of unsafety or safety, user friendliness, adverse events, and detection of clinical deterioration. One researcher (MW) performed a thematic content analysis to determine perceived positive and negative effects, and facilitators and barriers, which was critically reviewed by a second researcher (TB). Perceived positive and negative effects were presented according to the Donabedian framework for the quality of health care [36], which includes three main domains: structure, process, and outcome. Facilitators and barriers were divided into four domains: characteristics related to the patient, professional, intervention, and context [37].

## Results

### Demographics

**Figure 2:**
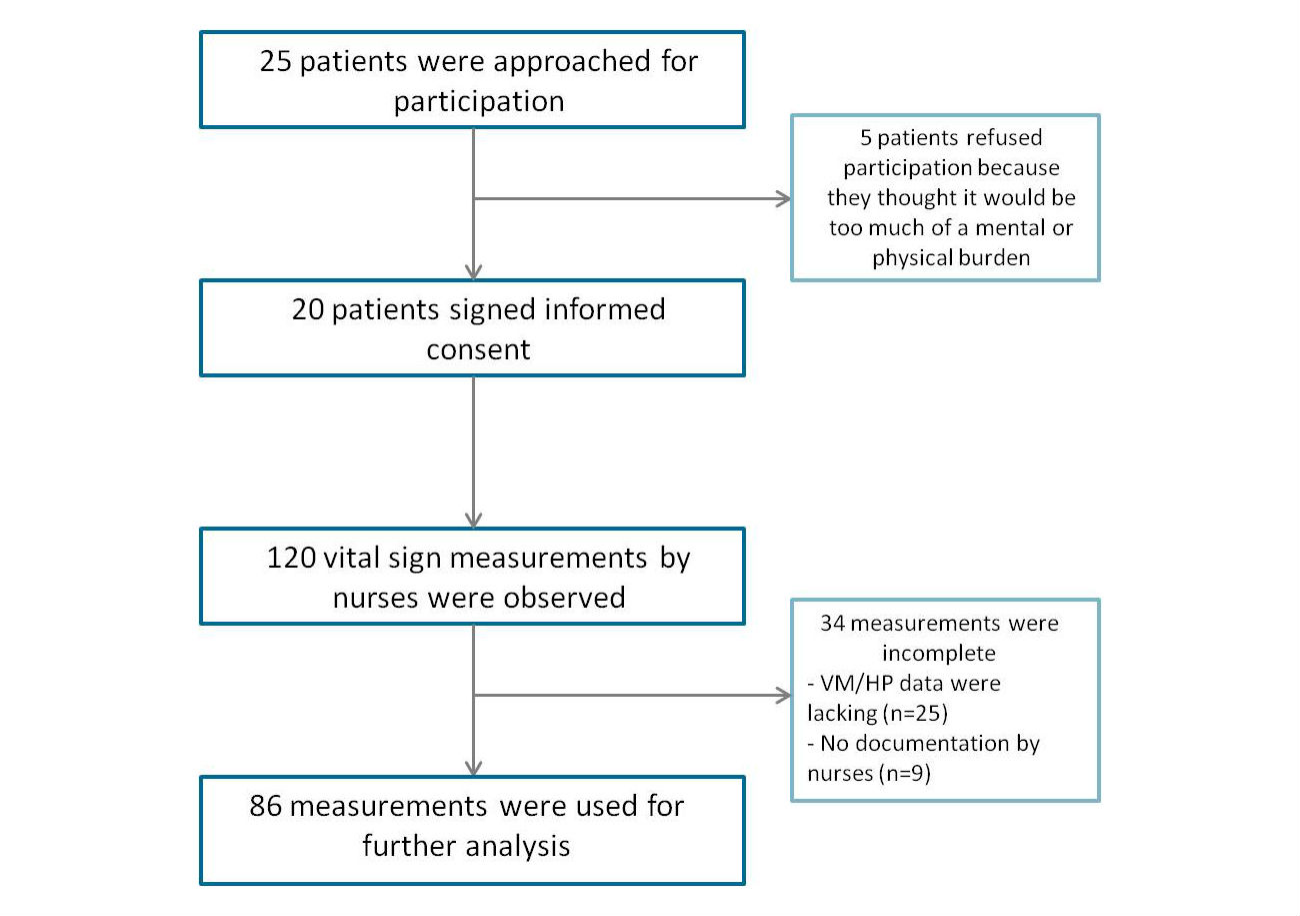
Included patients and vital sign measurements.

A total of 25 patients were invited, of which 20 participated in the study—10 patients at the surgical ward and 10 patients at the internal medicine ward. The other 5 patients refused participation because they thought it would be too much of a mental or physical burden (see Figure 2). The study population included 13 males and 7 females with a mean age (standard deviation, SD) of 49.9 (13.4) years, ranging between 33 and 82 years. At the surgical ward, most patients were admitted for an elective gastrointestinal operation. Patients at the internal medicine ward were admitted for several conditions such as sepsis, arthritis, and blood pressure control.

### Technical Feasibility

In total, 120 vital sign measurements by nurses were observed by the trained medical students (see Figure 2). In 40 measurements, one or more vital signs were missing. In 6 measurements, data were completed by consulting the EHR. As a result, 86 measurements were used for further analysis. For the remaining 34 measurements, VM and HP data were lacking (25 measurements), or vital signs were not documented by nurses (9 measurements). In 8 patients, data from the Toughbook was not available for further analysis due to accidental deletion of data; in 2 patients, no HP data were saved at the cloud server due to technical failures (eg, WiFi failures, disconnection between HP and its mobile device). In total, 742.8 hours of VM data and 1033.6 hours of HP data were collected; on an average 61.9 hours of VM and 57.5 hours of HP data were collected per patient.

**Figure 3:**
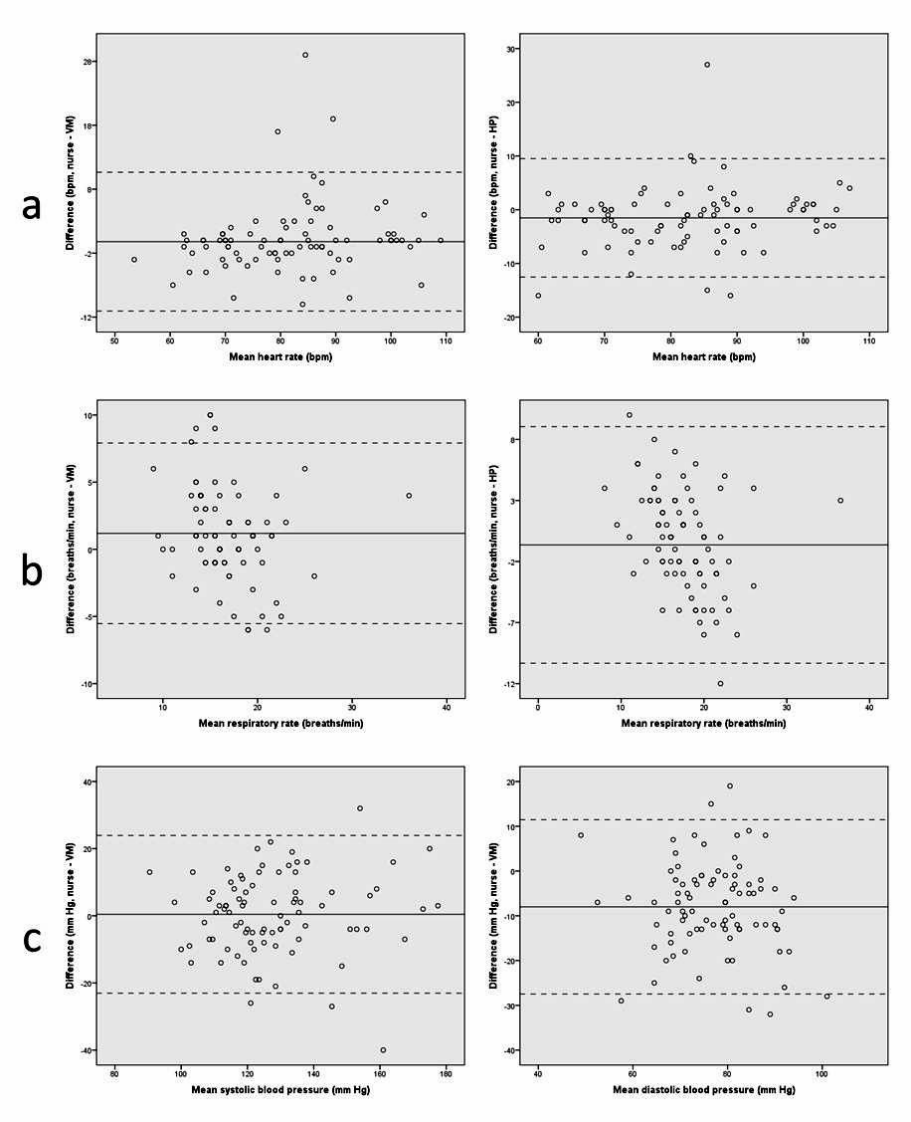
Bland-Altman plots: (a) heart rate (VM and HP), (b) respiratory rate (VM and HP), (c) systolic and diastolic blood pressure (VM). Dotted lines indicate mean difference and solid lines indicate limits of agreement.

**Figure 4:**
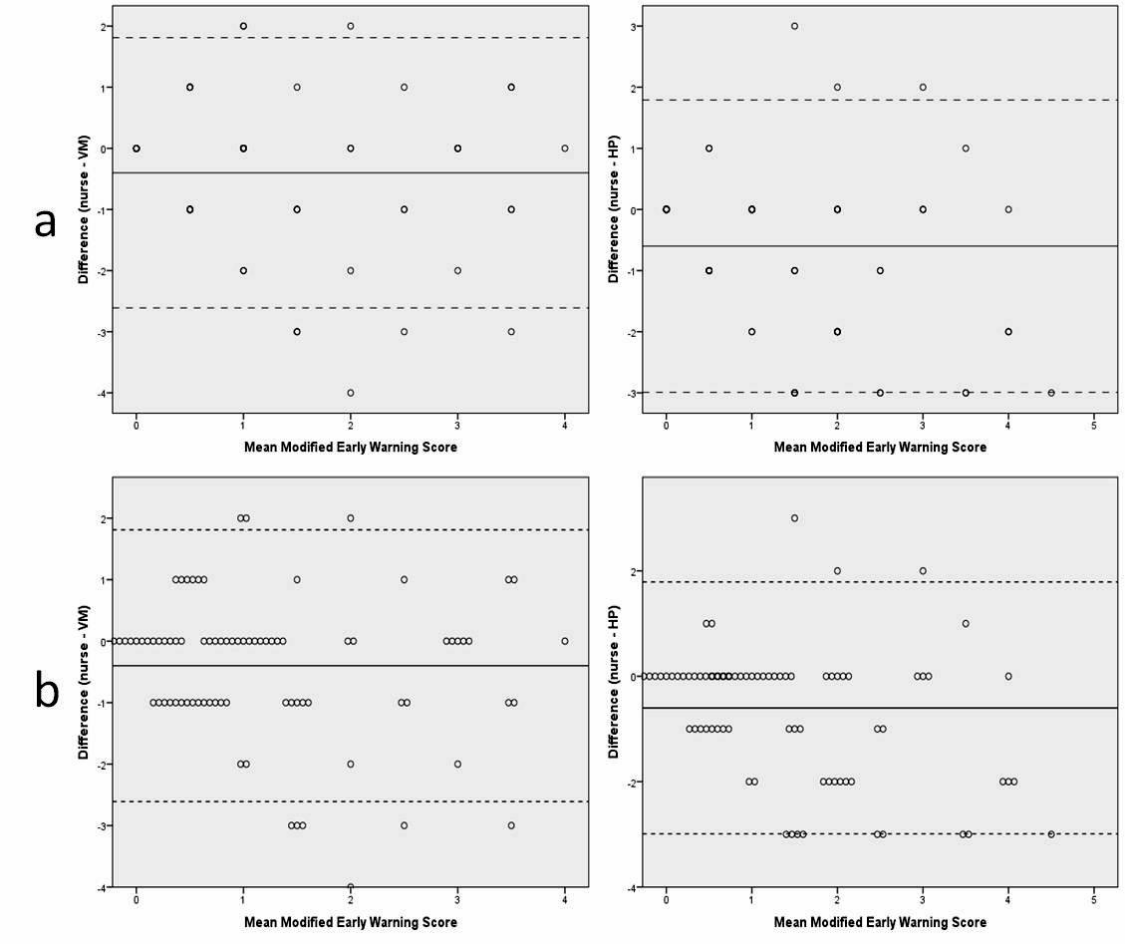
Bland-Altman plots showing modified early warning score: (a) VM and HP, (b) VM and HP (jittered). Dotted lines indicate mean difference and solid lines indicate limits of agreement.

### Vital Signs

Bland-Altman plots showing the mean of the two devices and the differences between the two devices (y-axis) with limits of agreement (1.96 SD) are displayed in Figures 3 and 4. Comparing the results for vital signs and MEWS score measured by nurses and VM, the mean differences were all within range with the predefined accepted discrepancies in Table 1, although wide limits of agreement were found (see Table 2). The largest discrepancy in the mean difference was found for diastolic BP. In 13 (15%) cases, the MEWS difference between nurse and VM was 2 points or higher, indicating important clinical differences between VM and nurse measurements (see Table 3). In four cases, this was related to differences in RR alone. In the remaining cases, the combination of RR and oxygen saturation, or RR and systolic BP caused the difference. Moreover, in six of these cases, VM measured a higher RR than nurses (range: 1-6 breaths/min), and in the four other cases, nurses measured a higher RR than VM (difference: 2-6 breaths/min). In the three remaining cases that resulted in a different MEWS, there was a difference in systolic BP (difference: 14 mm Hg) or oxygen saturation (difference: 1%-5%) between VM and the nurse. The mean differences between nurse measurement and HP were all in agreement with accepted discrepancies, although wide limits of agreement were found (see Table 2). In 23 (27%) cases, MEWS differed 2 or 3 points between HP and nurse measurements (see Table 3). In 17 cases, HP measured higher RR compared with nurses. In 16 cases, differences were in the range of 3 to 8 breaths/minute. However, in one case, nurses measured 16 breaths/minute and HP measured 42 breaths/minute, indicating possible measurement errors in HP. In the remaining six cases, nurses measured a higher RR than HP (difference: 4-12 breaths/min).

**Table 2:** ViSi Mobile and HealthPatch data in comparison with corresponding nurse measurements.

Vital signs	Nurse	ViSi Mobile		HealthPatch	
	Mean (SD)^j^	Mean (SD)	Mean difference (SD) versus nurse	Mean (SD)	Mean difference (SD) versus nurse
HR^f^ (beats/min)	81.81 (13.12)	81.62 (12.23)	−0.20 (5.54)	84.34 (12.24)	−1.52^c^ (5.63)
RR^g^ (breaths/min)	17.38 (3.89)	16.20 (4.57)	1.19^a^ (3.43)	18.02 (5.82)	−0.64 (4.94)
Saturation (%)	97.00 (96.00 to 98.00)^d^	97.00 (95.00 to 98.00)^d^	0.10 (1.65)	N/A^k^	N/A
Temperature (˚C)	37.01 ( 0.60)	33.61(1.25)^e^		34.16 (1.16)^e^	
BP^h^, systolic (mm Hg)	127.93 (19.33)	127.49 (18.68)	0.44 (11.99)	N/A	N/A
BP, diastolic (mm Hg)	73.17 (10.25)	81.17 (11.24)	−8.00^b^ (9.93)	N/A	N/A
MEWS^i^	0.99 (1.13)	1.38 (1.30)	−0.40^a^ (1.13)	1.59 (1.54)	−0.60^b^ (1.22)

^a^*P*=.002.

^b^*P*<.001.

^c^*P*=.01.

^d^Oxygen saturation was reported as median with interquartile range.

^e^Skin temperature.

^f^HR: heart rate.

^g^RR: respiratory rate.

^h^BP: blood pressure.

^i^MEWS: modified early warning score.

^j^SD: standard deviation.

^k^N/A: Not applicable.

### Artifacts

#### ViSi Mobile

In total, 306 artifacts were found, with a total time of 121 hours. In 111 (36.3%) of 306 artifacts, a trained medical student was present, and 86 of 111 (77.5%) were identified and reported. A cause was found in 82 (95.1%) of 86 artifacts. Almost 70% of all reported artifacts were caused by connection failure between Toughbook and VM. Other artifact causes were motion of the sensors due to patient movements (n=21, 25.6%) and required calibration of blood pressure (n=2, 2.3%). Over 74% of all artifacts lasted less than 5 minutes. Almost 20% lasted less than 1 hour, and approximately 7% lasted longer than 1 hour.

#### HealthPatch

In total, 648 artifacts were found in 18 patients, with a total time of 135 hours. More than 50% (n=354) of all artifacts lasted less than 1 minute and were excluded from further analysis. In the remaining 294 artifacts, a trained medical student was present in 60% (n=176) of the artifacts, and identified and reported the artifact in 53 (30%) cases. A cause was found in 24 (45%) artifacts such as HealthPatch losing skin contact (n=13, 54%), Bluetooth (n=4, 17%) or Wi-Fi problems (n=3, 13%), and patients leaving the ward without their mobile device (n=3, 13%). Around 43% of all artifacts lasted less than 5 minutes. Over 95% of all artifacts lasted less than 1 hour.

**Table 3:** ViSi Mobile (VM) and HealthPatch (HP) data in comparison with corresponding nurse measurements.

Vital signs	ViSi Mobile	HealthPatch
	Difference; nurse-VM (%)	Difference; nurse-HP (%)
HR^a^ (beats/min)	≤5: 71 (82.5)	≤5: 65 (75.6)
	6-10: 12 (14.0)	6-10: 16 (18.6)
	>10: 3 (3.5)	>10: 5 (5.8)
RR^b^ (breaths/min)	≤2: 50 (58.2)	≤2: 36 (41.9)
	3-5: 26 (30.2)	3-5: 31 (36.0)
	>5: 10 (11.6)	>5: 19 (22.1)
Saturation (%)	≤2: 76 (88.4)	N/A^e^
	3-4: 9 (10.5)	
	≥5: 1 (1.1)	
BP^c^ systolic (mm Hg)	≤5: 36 (41.9)	N/A
	6-14: 33 (38.4)	
	≥15: 17 (19.7)	
BP diastolic (mm Hg)	≤5: 27 (31.4)	N/A
	6-14: 40 (46.5)	
	15: 19 (22.1)	
MEWS^d^	−4: 1 (1.2)	−3: 9 (10.5)
	−3: 5 (5.8)	−2: 11 (12.8)
	−2: 4 (4.7)	−1: 13 (15.1)
	−1: 23 (26.7)	0: 47 (54.7)
	0: 40 (46.5)	1: 3 (3.5)
	1: 10 (11.6)	2: 2 (2.3)
	2: 3 (3.5)	3: 1 (1.2)

^a^HR: heart rate.

^b^RR: respiratory rate.

^c^BP: blood pressure.

^d^MEWS: modified early warning score.

^e^N/A: not applicable.

### Practical Usability

Evaluations were performed with all 20 patients, 7 relatives, and 4 nurses (see Table 4).

#### Perceived Positive and Negative Effects

##### Processes

One positive effect was identified in this dimension. Patients stated that nurses could keep an eye on the vital signs from a distance (n=3); this was also mentioned by one relative. No negative effects were identified.

##### Outcomes

Two positive effects were identified in this dimension. Eight patients and 66 relatives mentioned increased feelings of safety by being monitored continuously in comparison with the MEWS measurements by nurses only. A patient described:

Being monitored continuously is a very pleasant experience; I felt very safe.

Translated from Dutch

Earlier interventions were deemed possible in case of clinical deterioration (n=3). One negative effect was identified; one patient complained about having redness and itching while wearing the devices.

#### Facilitators and Barriers

##### Intervention

Seven facilitators were identified. Eight patients said they were not aware of the HP while it was attached to their chest. Other facilitators included not being restricted by the devices during daily activities such as bathing and putting on clothes (n=3), more freedom of movements compared with conventional devices (n=2), the small size of the HP (n=1), the good adhesive properties of the patches (n=1), and the invisibility of the devices under clothes (n=1). One patient described:

I have used a holter monitor at home several times. These devices are much smaller and they do not limit mobility to the same extent.

Translated from Dutch

One patient experienced great advantage of having an insight on his own vital signs. One barrier was noted 15 times. Patients mentioned that the VM wrist device was big or heavy (n=10); patches came off very quick (6 VM; 2 HP); VM had many cables (n=4); and VM had a short battery life (n=2).

##### Professional

Two facilitators and one barrier were identified in this domain. Two nurses stated that the patches did not lose skin contact while washing the patient, and one nurse said that it was very easy to attach the devices to the patient. One nurse mentioned that Wi-Fi connection was poor between Toughbook and the VM device.

#### Additional Findings

During the study, clinical deterioration was detected with the VM in one patient 3 days postoperatively after elective colorectal surgery. The device alerted the nurse who cared for the patient because he developed a tachycardia and tachypnea. This situation occurred between two regular measurements. He underwent relaparotomy after an anastomotic leak was confirmed by computer tomography.

**Table 4:** Users’ experiences.

Users’experience			Nurse	Patient	Relatives
**Perceived positive and negative effects**					
	**Processes^a^**				
		Nurse could keep eye on vital signs more easily		+	+
	**Outcomes**				
		Feelings of safety		+	+
		Earlier interventions		+	
		Adverse events (redness and itching)		−	
**Barriers and facilitators**					
	**Intervention**				
		Not aware of HP^b^		+	
		Small size of HP		+	
		Good adhesive properties		+	
		Not being restricted during daily activities		+	
		More freedom of movements		+	
		Invisibility under clothes		+	
		Better insight on own vital signs		+	
		VM^c^ wrist device too big/heavy		−	
		Patches came off very quickly		−	
		VM has too many cables		−	
		Short VM battery life		−	
	**Professional**				
		Good adhesive properties	+		
		Very easy to attach the devices	+		
		Bad Wi-Fi connection between VM and Toughbook	−		

^a^No positive or negative effects in the “Structure” or “Context” fields were found.

^b^HP: HealthPatch.

^c^VM: ViSi Mobile.

## Discussion

### Principal Findings

This study describes a unique approach in which we continuously measured vital signs on the ward using two recently released wireless devices. In general, data obtained by these devices correlated well with predefined accepted discrepancies and MEWS calculated on the basis of these devices correlated to a larger extent. Patients and nurses were mainly positive about the two devices. Both VM and HP are promising devices for continuous patient monitoring on the general ward. However, the number of artifacts should be reduced and the barriers mentioned by the users could be addressed to further improve both devices.

### Vital Signs

The largest discrepancy in mean difference was found in VM diastolic blood pressure, which is unlikely to be directly clinically meaningful since it is not a component of the MEWS. Additionally, clinical decisions are mainly based on systolic blood pressure and other vital signs. Wide limits of agreement were found for almost all vital signs and MEWS. Although more than 70% of all MEWS differed 0 or only 1 point between devices and nurse measurements, larger differences in MEWS were found in a few cases, which may have important clinical consequences (eg, additional diagnostic procedures or change in treatment). In most of these cases, VM and HP measured a higher RR when nurses did not. Although most differences between nurse and device measurements were small (<5 breaths/min), in one case, difference between nurse and HP measurements was large (26 breaths/min). These findings are important as abnormal RR has been shown to be an important predictor of cardiac arrest [38] and an indicator of sepsis, pneumonia and respiratory depression [39]; therefore, it could under- or overestimate a clinical condition of a patient. Inaccurate RR measurements by nurses could explain the discrepancy. Direct measurement of RR is usually done by visually observing chest movement or by manual observations. Reproducibility may be limited by significant interobserver variability [40]. Conversely, ECG-derived RR measurements by HP and VM may be inaccurate. In case of HP, RR is estimated by ECG using the respiratory sinus arrhythmia method, which derives RR from HRV. Since this method has some limitations, an accelerometer was added to measure RR more accurately [41]. In VM, RR is derived from impedance pneumography, measuring respiratory volume and rate through the relationship between respiratory depth and thoracic impedance rate [42]. ECG-derived RR may not be accurate when there is excessive patient motion or during lower respiratory rates [43,44]. More research is required to gain a deeper insight in the different methods of measuring RR by devices and nurses.

### Artifacts

Most reported that VM artifacts concerned connectivity failure between VM and Toughbook. This was caused by a restricted Wi-Fi connection of approximately 15 meters between VM and Toughbook, which explains why more artifacts were found in mobile patients. These artifacts were not deemed relevant since more stable Wi-Fi connections, such as by using multiple access points and 5 Ghz networks, would be used to implement VM in a hospital setting. This would also facilitate continuous monitoring during patient transport between different wards. However, it is important to consider that a wireless connection can always fail, thus proper backup power and Internet connections are always demanded. Most HP artifacts were of unknown cause. However, most artifacts lasted less than one hour and were self-limiting.

Although HP could not measure all vital signs that are currently used to monitor patients and to calculate the MEWS, it may still provide more patient data than interval measurements by nurses, resulting in a more continuous dataflow and more specific trends. This may be of significance, in particular, since literature shows important lack of documentation of vital signs before a life-threatening event [27]. Besides that, several studies show that HR and RR change significantly before ICU transfer, cardiac arrest, and mortality and therefore, HP can have a valuable contribution to the prediction of life-threatening events [24,27].

### Practical Usability

The majority of patients, relatives, and nurses were positive about VM and HP. Whereas HP is able to administer vital signs in real time to patient’s mobile phone, VM shows vital signs in real time on the wrist device; these devices could therefore increase insight on patient’s health status and potentially influence their behaviors [45,46]. Although patients mentioned that the VM wrist device was heavy and VM consisted of many cables, they were not restricted during daily activities or mobility. This is important as hospitalized patients benefit from mobility, resulting in increased recovery and reduced risk of complications [47,48]. Another benefit of VM and HP is that nurses are able to see patients’ vital signs from a distance. A review by Ulrich et al [49] has shown that sleep deprivation in patients is a common problem that is associated with hindrance of the healing process and an increase in morbidity and mortality. Using VM and HP, patients could continue sleeping during the night and did not have to be disturbed by vital sign measurements.

Possible negative aspects of continuous monitoring should also be taken into consideration. Wearable devices generate a large quantity of data each day. The workload of nurses and physicians withholds them from inspecting all these data, which means that the predictive value of continuous monitoring is lost [17]. Validated devices are available to process all these data and to send an alert when patient’s vital signs drop out of normal ranges. A large number of alerts and even false-positive alerts could cause alarm-fatigue in nurses [17,50]. Algorithms using machine learning could be utilized to reduce false-positive alarms [51-53]. The VM battery has a battery life of 12 to 14 hours, which means that nurses have to change batteries twice a day. This might outweigh the fact that nurses no longer need to perform the standard MEWS measurements three times a day.

### Comparison With Prior Work

A few studies about continuous monitoring at the general ward have been published. A wireless sensor was successfully used in pregnant women in an inpatient obstetric unit, which was able to monitor HR, RR, and temperature [30]. Recently, the SensiumVitals digital patch was tested in hospitalized patients [54]. This patch is able to measure HR and RR and was compared with a commonly used clinical monitor. A satisfactory agreement, comparable with the result in our study, was shown. The drawback of the study design was that the patients were monitored for only 2 hours, which prevented the authors from detecting trends in vital signs and lowered predictive value. The use of an implantable device for continuous monitoring has been described in the ambulatory setting. Abraham et al [55] described the use of a wireless implantable hemodynamic monitoring system in heart failure patients, which has shown to reduce hospitalization. Wireless technology systems in which patients measure vital signs at home have been described, such as for patients with chronic obstructive pulmonary disease [56], patients with hypertension [57], and patients with diabetes mellitus [58,59]. These systems were often well received by patients and health care providers, showing improvement of blood values such as glucose [58,60], patients’ disease management [56,61], and better connection between the patient and the health care provider [59]. Particularly, the HP might be suitable for home monitoring, although its current version lacks the possibility to measure all vital signs. Though VM measures all vital signs, its size and cables might demand much from patients to enable monitoring at home.

### Strength and Limitations

An important strength of the study is that we were able to monitor patients in a clinical setting instead of healthy participants in controlled settings. The study had a small sample size, and we missed some VM and HP data, particularly since VM data of 8 patients were automatically deleted from the Toughbook and could not be used for artifact analysis. This was due to wrong Toughbook settings and was changed with support from the manufacturer. The VM vital signs observed by students were used for the comparison with nurse measurements, and we were therefore able to draw conclusions about the feasibility of both VM and HP. However, data saturation in patient, nurse, and relative interviews may not have been reached. Selection bias could have occurred as not all patients who were approached did agree to participate. A further limitation of VM and HP is that both devices measure skin temperature instead of body temperature. Although it is not yet clear whether or not all vital signs are necessary for proper clinical judgment of ill patients, an algorithm should be developed to convert skin temperature into body temperature.

### Conclusions

The VM and HP are promising devices for wireless continuous patient monitoring in the hospital and were very well received by both patients and nurses. The frequency and duration of artifacts should be reduced and the barriers mentioned could be addressed to further improve VM and HP. An ongoing follow-up study focuses on the different effects of VM or HP compared with routine MEWS measurements on patient comfort and safety and nurse workload, and on early detection of deterioration. Future studies should focus on the effect of continuous monitoring on clinical outcome.

## Appendix

Multimedia Appendix 1 Modified Early Warning Score (MEWS); A: alert, V: response to speaking, P: response to pain, U: no response.

## Abbreviations

BP: blood pressure
CE: Conformité Européenne
ECG: electrocardiogram
EHR: electronic health records
EWS: early warning scores
FDA: US Food and Drug Administration
HP: HealthPatch
HR: heart rate
ICU: intensive care unit
MEWS: modified early warning score
RR: respiratory rate
SD: standard deviation
VM: ViSi Mobile
