# Correction: Continuous Monitoring of Vital Signs Using Wearable Devices on the General Ward: Pilot Study

**DOI:** 10.2196/31899

**Published:** 2021-07-16

**Authors:** Mariska Weenk, Harry van Goor, Bas Frietman, Lucien JLPG Engelen, Cornelis JHM van Laarhoven, Jan Smit, Sebastian JH Bredie, Tom H van de Belt

**Affiliations:** 1 Radboud University Medical Center Department of Surgery Nijmegen Netherlands; 2 Radboud University Medical Center Radboud REshape Innovation Center Nijmegen Netherlands; 3 Radboud University Medical Center Department of Internal Medicine Nijmegen Netherlands

In “Continuous Monitoring of Vital Signs Using Wearable Devices on the General Ward: Pilot Study” (JMIR Mhealth Uhealth 2017;5(7):e91), one error was noted.

In the originally published manuscript, an incorrect ORCID number was listed for author Jan Smit. This ORCID number has now been removed.

The correction will appear in the online version of the paper on the JMIR Publications website on July 16, 2021, together with the publication of this correction notice. Because this was made after submission to PubMed, PubMed Central, and other full-text repositories, the corrected article has also been resubmitted to those repositories.

