# Supplementary figures and images for: Continuous Monitoring of Vital Signs Using Wearable Devices on the General Ward: Pilot Study
Source: JMIR Mhealth Uhealth. 2017 Jul 5;5(7):e91. doi: 10.2196/mhealth.7208 (PMC5517820; doi:10.2196/mhealth.7208)

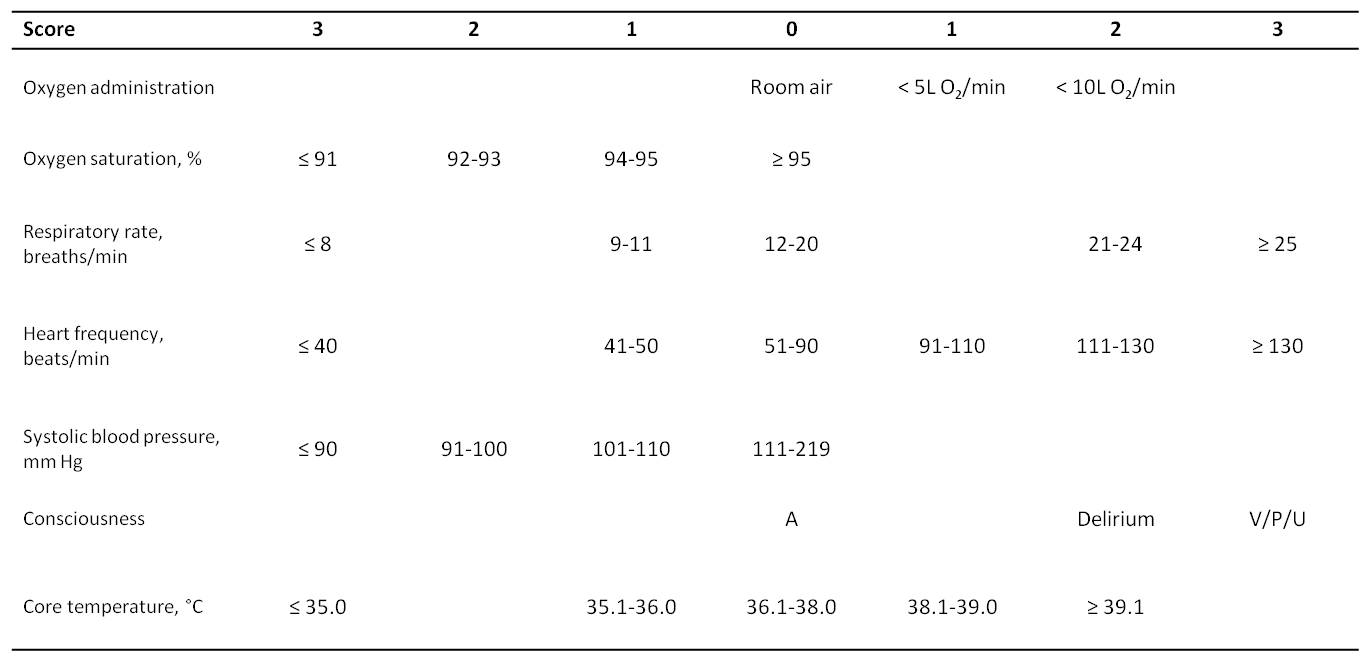

Supplement: Multimedia Appendix 1 [file mhealth_v5i7e91_app1.jpg]
